# Multi-Step Fractionation of New High Stearic Sunflower Oils

**DOI:** 10.3390/foods15101784

**Published:** 2026-05-18

**Authors:** Joaquín J. Salas, Enrique Martínez-Force, Miguel A. Bootello, Mónica Venegas-Calerón

**Affiliations:** Instituto de la Grasa—CSIC, Ctra Utrera Km 1, Building 46, 410013 Sevilla, Spain; emforce@ig.csic.es (E.M.-F.);

**Keywords:** high-stearic sunflower, oil interesterification, oil fractionation, stepped fractionation, stearin, olein, melting profile

## Abstract

Fractionation plays a key role in the manufacture of specialty fats, particularly when applied to tropical oils characterized by a high content of saturated fatty acids, such as palm and coconut oils. This technique is based on the selective crystallization of triacylglycerols (TAGs) with higher saturation, followed by separation of the solid (stearin) and liquid (olein) phases to obtain fractions with increased solid content. The approach has also been extended to sunflower oils enriched in stearic and oleic acids. Here, we further examine the fractionation of high-stearic sunflower oils. Newly developed oil varieties with elevated stearic acid levels (20 to 22%) and varying oleic-to-linoleic acid ratios were evaluated. Additionally, the feasibility of applying a multistep fractionation strategy, similar to that used for palm oil, was assessed. Fractionation was successfully achieved under all studied conditions, and the physico-chemical properties of the resulting fractions were analyzed. The novel two-step process increased stearin recovery to overall yields above 50%, producing two distinct fractions: a solid fat with progressive melting behavior, and a mid-fraction enriched in disaturated TAGs with a faster melting profile, both of which may offer promising applications in a wide range of food formulations. The implications of these findings for the design and implementation of future industrial-scale fractionation processes are discussed.

## 1. Introduction

Over the last few decades, several conventional oilseed crops have been altered by mutagenesis or genetic engineering with the aim of increasing their saturated fatty acid content. This strategy has been applied to species such as sunflower, rapeseed, and soybean [1,2,3]. Within these modified crops, high-oleic high-stearic (HOHS) sunflower varieties are particularly noteworthy, as they yield oils enriched in stearic acid while preserving a high proportion of oleic acid. These characteristics make HOHS sunflower oil a promising non-transgenic alternative to tropical fats that can be cultivated under temperate climatic conditions [4]. Nevertheless, the metabolic constraints of sunflower limit the formation of highly desaturated triacylglycerols (TAGs) in HOHS oils, promoting instead the accumulation of mono- and triunsaturated (UUU) TAG species [5]. As a consequence, HOHS sunflower hybrids grown in different environments typically produce oils containing between 16% and 20% stearic acid [4]. At these saturation levels, HOHS oils behave as highly stable liquid oils with higher cloud points than conventional or high-oleic sunflower oils, although their melting profiles are still unsuitable for direct application in plastic fats or confectionery formulations.

Previous studies have addressed the fractionation of HOHS sunflower oils [6,7]. Using solvent fractionation, these oils can yield solid fractions or stearins with elevated solid fat content, suitable for the production of cocoa butter equivalents and chocolate-type products with desirable functional properties. Dry fractionation has also been explored; however, this process presents greater technical challenges compared with palm oil fractionation. HOHS sunflower oil exhibits slower and less efficient crystallization behavior, making crystal seeding necessary to achieve a stable and reproducible process. Furthermore, the presence of naturally occurring wax esters (WEs) in sunflower oil interferes with crystal growth and separation, delaying crystallization and causing clogging of filtration fabrics during the concentration of crystallized TAGs. For this reason, a preliminary partial dewaxing step is usually required before fractionation [7]. This process results in a stearin fraction enriched in disaturated (SUS) TAGs, which shows potential for use as a plastic fat, while the corresponding olein fraction is dominated by monounsaturated TAGs and constitutes a highly stable liquid oil suitable for frying applications.

The distribution of saturated fatty acids in these oils is symmetric, which means that saturated fatty acids tend to occupy the *sn*-1 and *sn*-3 positions of the TAG, with the *sn*-2 position occupied by an unsaturated fatty acid. Enzymatic interesterification (EIE) has been widely investigated as a versatile tool to tailor lipid structure and functionality under mild conditions, with applications ranging from trans-fat replacement to the design of structured lipids [8]. Over the past two decades, research has progressed from proof-of-concept studies to more controlled processes using immobilized lipases, enabling improved regioselectivity, scalability, and industrial feasibility. Recent work has focused on optimizing reaction conditions, enzyme stability, and integration with downstream processes such as fractionation [9,10,11]. Despite these advances, challenges remain in controlling product distribution and maximizing yields of targeted fractions. In this context, the present study builds on prior developments by combining EIE with fractionation to enhance stearin recovery and functionality. A recent study indicated that when this distribution was altered through enzymatic interesterification, the fractionation process was facilitated, increasing the yield of stearin and its solid fat content (SFC) without requiring prior dewaxing or crystal seeding [12]. In the present article, these results were taken as a starting point, and various possibilities of combining EIE of HSHO oils followed by fractionation were explored. In this context, the effect of the degree of interesterification on fractionation was examined, investigating the minimum threshold necessary to facilitate the process and its impact on the resulting solid fractions. On the other hand, the EIE process induces the formation of trisaturated (SSS) TAGs, which, although they increase the SFC, may impart undesirable properties. In this study, a stepwise fractionation procedure was tested for these oils, consisting of successive crystallizations at lower temperatures. This made it possible to obtain a stearin fraction rich in SSS TAGs and a medium fraction enriched in SUS, using a procedure similar to that employed for palm oil fractionation [13,14]. This would enable the versatile production of stearins rich in SSS TAGs, suitable for the production of stearic-rich fats with different melting profiles through interesterification with liquid oils. Intermediate fractions resulted in fats with sharper melting profiles, which were comparable to those of confectionery fats. In all cases, the fats were stearic acid based, which is considered a healthier fatty acid compared to palmitic acid or short-chain fatty acids. Therefore, this new process opens the door to the production of a new line of healthier fats. Both types of fractions were characterized in terms of composition and physical properties, and the potential scalability of the process was discussed based on the results obtained.

## 2. Experimental

### 2.1. Oils Used

In this study, two high-stearic sunflower oils were used. The first oil contained 19.5% stearic acid within a high-oleic background and was designated HO20. The second oil exhibited a higher total saturated fatty acid content, with 22% stearic acid, together with an increased level of linoleic acid (medium-linoleic background), and was designated ML22. Initially they were non-interesterified (non-IE) oils. The same oils, after being subjected to enzymatic interesterification, were referred to as HO20/EIE and ML22/EIE. These oils were produced from seeds grown in the experimental orchard of the Instituto de la Grasa, which were extracted using a Tubby press. The resulting oil was then neutralized and bleached, producing an RBD-quality oil that was used in the experiments described in this work (AV < 0.5%, PV < 10). The density and viscosity of these oils at 25 °C was not very different from that of a high-oleic sunflower oil and stayed at about 0.92 g/cm^3^ and 75 mm^2^/s. Enzymatic interesterification reactions were performed using Lipozyme TL (Novozymes, Copenhagen, Denmark) following a procedure described later.

### 2.2. Fatty Acid Composition

The fatty acid profiles of all oils and derived fractions were determined after conversion of the lipids to fatty acid methyl esters (FAMEs), followed by gas chromatographic analysis with flame ionization detection (GC-FID). For this purpose, samples (5–10 mg) were subjected to acid-catalyzed transesterification at 80 °C for 1 h using 2 mL of methanol containing 2% sulfuric acid and 10% toluene. After completion of the reaction, the resulting FAMEs were recovered by extraction with 2 mL of heptane. Chromatographic analysis was performed on an Agilent 7890 gas chromatograph (Agilent, Palo Alto, CA, USA) fitted with a Supelco SP-2380 capillary column (30 m × 0.25 mm i.d., 0.2 μm film thickness; Supelco, Inc., Bellefonte, PA, USA), operated under isothermal conditions at 180 °C. The injector and flame ionization detector temperatures were set at 220 °C. Hydrogen was used as the carrier gas, applying a pressure program from 80 to 100 kPa (flow from 1.8 to 2.3 mL/min) at a rate of 10 kPa·min^−1^, with a split ratio of 40:1. The samples were prepared to obtain a final concentration of 5 mg/mL of methyl esters, from which 1 µL was typically injected. No make-up gas was used, and hydrogen was employed as the carrier gas. The air flow in the detector was set at 20 mL/min. Prior to each series of analyses, a standard of known composition was injected to verify column performance and system response. The analyses were completed within a total run time of 10 min. A compositional analysis was performed by integrating all the peaks in the chromatogram and expressing the results as percentages of the integrated peak areas. Individual fatty acids were identified by comparison of their retention times with those of certified reference standards consisting of commercial methyl esters (Merck, Darmstadt, Germany).

### 2.3. Triacylglycerol Composition Analysis

The TAG profile of the oils was determined by gas chromatography, following an analytical approach based on that previously reported by Bootello et al. [2]. Prior to analysis, samples were diluted in hexane and injected directly into an Agilent 8890 gas chromatograph (Agilent, Palo Alto, CA, USA) fitted with a Quadrex Aluminum-Clad 400-65HT capillary column (30 m × 0.25 mm i.d., 0.1 μm film thickness; Quadrex, Woodbridge, CT, USA). Hydrogen served as the carrier gas. The injector and flame ionization detector were both operated at 350 °C, while the oven temperature was maintained at 320 °C. A pressure program ranging from 70 to 120 kPa was applied during the chromatographic run at a rate of 10 kPa·min^−1^ (flows from 1.5 to 2.8 mL/min), and a split ratio of 40:1 was used. The samples were prepared to obtain a final concentration of 5 mg/mL of triacylglycerols (TAGs), from which 1 µL was typically injected. No make-up gas was used, and oxygen was employed as the carrier gas. The air flow in the detector was set at 20 mL/min. Prior to each series of analyses, a standard of known composition was injected to verify column performance and system response. The analyses were completed within a total run time of 30 min. TAG species were identified and quantified according to the procedure described by Fernández-Moya et al. [15], reporting the results as relative percentages based on their integrated areas and the response factors reported by Carelli and Cert [16]. Peak assignment was performed based on the retention times of known standards and the carbon number of the expected compounds. When required, commercial triacylglycerol standards supplied by Larodan (Solna, Sweden) were employed for peak assignment.

### 2.4. Enzymatic Interesterification of Oils

Enzymatic interesterification was performed using immobilized Lipozyme TL (Novozymes, Bagsværd, Denmark). Before initiating the reaction, the biocatalyst was preconditioned with the same oil by contacting 50 g of enzyme with 500 mL of HOHS sunflower oil at 70 °C for 30 min under vacuum, applying agitation in a rotary evaporator in three successive cycles. After conditioning, the enzyme was recovered by filtration through a Büchner funnel and kept stored at 5 °C with a dissicant. The oils were preconditioned by applying a vacuum at 80 °C in a rotary evaporator to remove traces of moisture. Preconditioned oils were then used for enzymatic interesterification experiments, which were conducted in a 1 L reactor at 70 °C for 24 h under a nitrogen atmosphere, with continuous stirring to maintain uniform suspension of the enzyme particles. The catalyst, added at a concentration of 0.5% (*w*/*w*), was introduced once the oil had reached the desired reaction temperature. At the end of the reaction period (10–16 h), the interesterified oil was separated from the enzyme by Büchner filtration, and the recovered enzyme was either reused or stored at 5 °C for later use. Under these operating conditions, the interesterification proceeded to completion and the increase in acidity value did not exceed one unit. Oils exhibiting an increase of 1.0 unit or higher in acidity value were subjected to a subsequent neutralization step.

### 2.5. Dry Fractionation of Oils

Oil fractionation was carried out at laboratory scale in a 1 L crystallization vessel. Initially, the oil was introduced into the crystallizer and heated to 50 °C to eliminate any preexisting crystalline structures. Rapid cooling to 35 °C was then applied using the maximum cooling capacity of the thermostatic bath. Subsequently, the temperature was gradually lowered to the nucleation point over a period of 2 h. Once this temperature was reached, the system was held isothermally for 3 h to promote nucleation. Crystal growth was then induced by applying a further controlled cooling ramp to the final crystallization temperature over 2 h (see Supplementary Figure S1). The mixture was kept at that temperature for at least 16 h. Stirring was kept to a minimum to maintain a homogeneous temperature within the crystallizer (approximately 30 rpm). Thus, the fractionation conditions will be referred to throughout this article using two temperatures separated by a slash; the first indicates the nucleation temperature, and the second the final crystal formation temperature. Once the slurry was formed, a volume of 50 mL was loaded into a filtration high-pressure stainless-steel filtration device, where the olein was vacuum filtered using a high-pressure-tested filtering cloth supplied by Desmet Ballestra. The stearin retained in the filtration chamber was subjected to squeezing by applying pressure up to 9 bar to eliminate olein trapped within the stearin cake. This system (Supplementary Figure S2) closely simulates the industrial fractionation process on a laboratory scale. Three to five filtrations were applied in each fractionation, and the fractions obtained were then characterized.

### 2.6. Differential Scanning Calorimetry (DSC)

The thermal behavior of the oil fractions, including melting and crystallization characteristics, was evaluated by differential scanning calorimetry (DSC) using a Q2000 V23.5 instrument (TA Instruments, New Castle, DE, USA). Prior to analysis, the calorimeter was calibrated with indium, azobenzene, and undecane standards. Approximately 6–7 mg of each oil or fat sample was weighed into Tzero aluminum pans, which were hermetically sealed; an empty pan was used as the reference. Sample and pan masses were determined using an electronic microbalance (Sartorius M2P; Sartorius AG, Göttingen, Germany). For the determination of melting behavior, any previous crystalline structure was eliminated by heating the samples to 80 °C and holding for 5 min. The samples were then cooled to −80 °C using a controlled cooling ramp and maintained at this temperature for 5 min. Subsequently, melting curves were recorded by heating the samples from −80 °C to 90 °C at a rate of 5 °C·min^−1^ while monitoring heat flow. Crystallization behavior was assessed in a separate run by first heating the samples to 90 °C for 5 min to erase thermal history, followed by cooling from 90 °C to −80 °C at 5 °C·min^−1^. In both analyses, peak temperatures and transition enthalpies were determined from the resulting thermograms.

### 2.7. Statistical Analysis

All analytical determinations were conducted in triplicate or quadruplicate, and the composition of the initial oils was reported as the mean of three technical replicates. Fractionation experiments were likewise performed in triplicate, and results are presented as mean values ± standard deviation obtained from independent runs. The evaluated parameters included mixture viscosity, stearin yield, solid fat index (SFI), and the compositional characteristics of the resulting fractions. For statistical comparison of means, the final fractionation temperature was considered the treatment factor, and data were analyzed by one-way analysis of variance (ANOVA), followed by Tukey’s post hoc test, with statistical significance set at *p* < 0.05. Differential scanning calorimetry (DSC) measurements were also carried out in triplicate, and values corresponding to stearin samples from different experiments were averaged, with the associated standard deviations reported. The analyzed DSC parameters included melting peak temperatures, crystallization onset, and melting intervals. All statistical analyses were performed using IBM SPSS Statistics version 27.

## 3. Results and Discussion

High-stearic sunflower oils are produced in sunflower plants that exhibit a deficiency in stearic acid desaturation. This function is essential for the plant, and its complete suppression would be lethal. Therefore, different high-stearic lines have been developed, exhibiting varying stearic acid contents ranging from 11% to 35%. In addition to the stearic acid content, the physicochemical characteristics of the oil are also influenced by the genetic background or relative content of oleic and linoleic acids. All these factors affect the fractionation process of these oils. In previous studies, oils with moderate stearic acid content (14–16%) and high oleic acid content were fractionated. However, newly developed hybrids have significantly increased stearic acid content, reaching stable levels around 20% in high-oleic backgrounds and surpassing this percentage when there are higher levels of linoleic acid.

In the present study, we investigated the efficient fractionation of two high-stearic–high-oleic sunflower oils, both with elevated stearic acid contents but differing in their proportions of linoleic acid, with the aim of understanding how these parameters influence the efficiency of the fractionation process. The first oil contained 19.5% stearic acid within a high-oleic background (67% oleic acid, Table 1 and Table 2), and linoleic acid (18:2) below 5%. This oil was designated as HO20. The second oil exhibited a higher stearic acid content of 22.1%, along with a greater proportion of linoleic acid (13.9%, Table 1 and Table 2), and was designated as ML22. The triglyceride composition of these oils is shown in Table 2. Within the TAG profile, the proportion of high-melting-point TAGs, specifically SUS and SSS TAGs, is particularly relevant, as these will crystallize during the fractionation process and thus enable the production of fats with a higher solid content. Due to the natural distribution of saturated fatty acids in sunflower oil, these tend to accumulate at the *sn*-1 and *sn*-3 positions of TAGs, meaning that high-stearic oils are rich in SUS TAGs (Table 1) and contain only trace amounts of SSS TAGs. When these oils are subjected to enzymatic interesterification, the distribution of saturated fatty acids becomes randomized, leading to an increase in SUS TAGs, from 16.4% to 17.1% in HO20 and from 15.7% to 18.1% in ML22. Moreover, SSS TAGs appear at levels of 1.9% and 2.7%, respectively, resulting in a total content of high-melting-point TAGs of 19% and 20.8%. The increase in these TAG species, as well as their new distribution, favors and facilitates the fractionation of these oils, as previously demonstrated [12].

The fractionation of high-stearic oils can be performed by lowering the temperature after a structure destruction step at 50 °C. The initial cooling or nucleation step is carried out gradually with gentle stirring at a temperature where the oil begins to crystallize, and it is maintained for a period to allow the formation of stable crystals. Once formed, a crystal growth stage begins during which the temperature is progressively decreased until a final setpoint, which is then maintained until the end of the process. The cooling profiles used for the fractionation of oils HO20/EIE and ML22/EIE are shown in Supplementary Figure S1 at the aforementioned temperatures.

Previous studies have reported results on HOHS oils with stearic acid content between 14% and 16% [6,7]. However, advances in the development of new mutations and their expression in different genetic backgrounds have resulted in lines with a higher stearic acid content and elevated levels of linoleic acid. This variability in the initial feedstock composition is not observed in other saturated fat sources, such as palm, palm kernel, or coconut oils. Therefore, it is necessary to investigate how the fractionation process must be adapted to changes in the composition of the initial oil and also to study how the physicochemical properties of the resulting products are affected. Until now, a single-step fractionation has been applied to obtain a stearin fraction and an olein fraction from the initial oils. This fractionation strategy was applied to the HO20/EIE and ML22/EIE oils. Initially, a single-step fractionation strategy was explored with the aim of maximizing stearin yield. Different combinations of nucleation and crystal growth temperatures were tested, as described in Supplementary Figure S1. The combinations of 20 °C/16 °C and 22 °C/12 °C for HO20/EIE and ML22/EIE oils, respectively, were particularly effective. Higher final temperatures resulted in lower recovery of high-molecular-weight TAGs, whereas lower temperatures yielded slurries that were too viscous for effective filtration and squeezing. The compositions of the resulting fractions are presented in Table 3. The minimum temperatures at which effective fractionation occurred were 16 °C for HO20 and 12 °C for ML22. In particular, despite its higher content of stearic acid, ML22 oil could be fractionated at lower temperatures than its high-oleic counterpart (HO20), because the crystallized slurries were less viscous and exhibited filtration and squeezing issues at higher temperatures.

**Figure 1:**
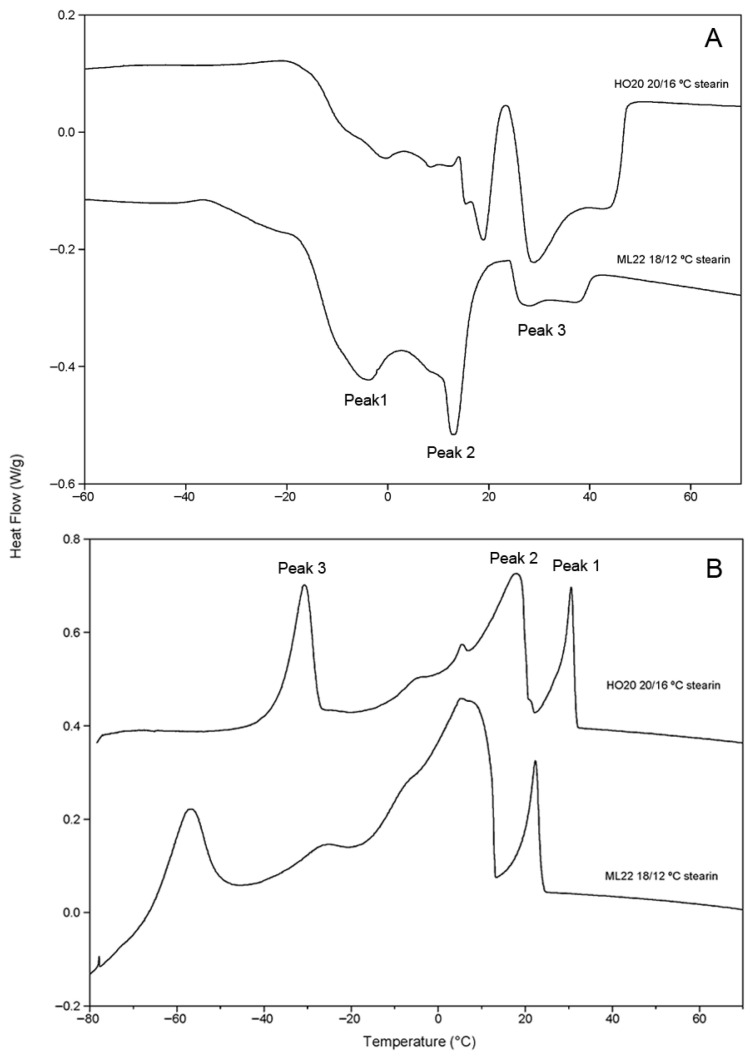
Melting (**A**) and crystallization (**B**) curves of stearins produced by single-step fractionation of high-stearic sunflower oils HO20 and ML22 at 20 °C/16 °C and 20 °C/12 °C, respectively. Exo up.

**Figure 2:**
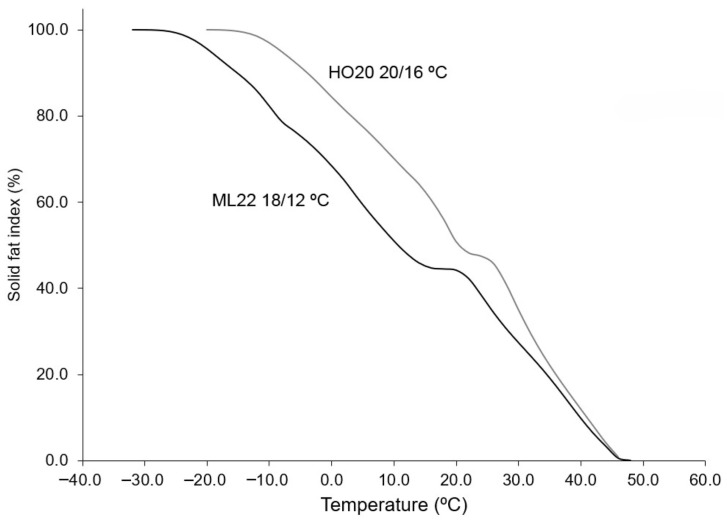
Solid fat index (SFI) curves of stearins resulting from single fractionation of HO20 (grey) and ML22 (black). The records were obtained by continuous integration of DSC melting curves.

The compositions of the resulting fractions are detailed in Table 3. In both cases, the resulting stearins showed SSS TAG levels of 9.2% and 8.0%, respectively. The proportion of SUS TAGs was also considerable, accounting for 38.5–39.3% of the stearin fraction. When comparing the two oils, the most notable difference was in the yield of stearin: 25.7% for HO20 versus 38.9% for ML22. This correlates well with olein compositions, where the depletion of SUS-type TAGs was more pronounced in ML22 than in HO20. This is due to the ability to fractionate at lower temperatures in ML22 oil, despite its higher stearic acid content. Furthermore, the resulting stearins had levels of stearic acid exceeding 30%. The physical properties of these stearins were characterized using differential scanning calorimetry (DSC), with their melting and crystallization profiles shown in Figure 1. Both fats exhibited similar melting profiles, with three main peaks corresponding to different TAG families (SSS, SUS + SSU, SUU + USU, and UUU). The highest melting peak corresponded to SSS-type TAGs and was more prominent in HO20 stearin obtained at 16 °C, due to its higher proportion of these compounds. Stearins HO20 20 °C/16 °C and ML22 18 °C/12 °C showed crystallization onsets at 33.2 °C and 26.8 °C, respectively. Relevant data from the DSC curves are presented in Table 4, where it can be observed that both fats exhibit wide melting ranges, spanning 70.9 °C for HO20 and 78.4 °C for ML22. The continuous integration of the melting curves allows for estimation of the SFI at different temperatures for both fats, as shown in Figure 2. The figure illustrates the typical progressive melting profile of these fats, with the solid fat index being consistently higher in the case of the HO20/16 stearin, likely due to its higher content of SSS and oleic components. These melting profiles are suitable for bakery shortenings and the formulation of margarines and spreads [17,18]. Additionally, due to their high content of stearic acid, they could be used to produce structured fats by interesterification. On the other hand, they also possess elevated levels of SSS TAGs, which is undesirable in certain formulations where graininess or waxiness must be avoided.

**Figure 3:**
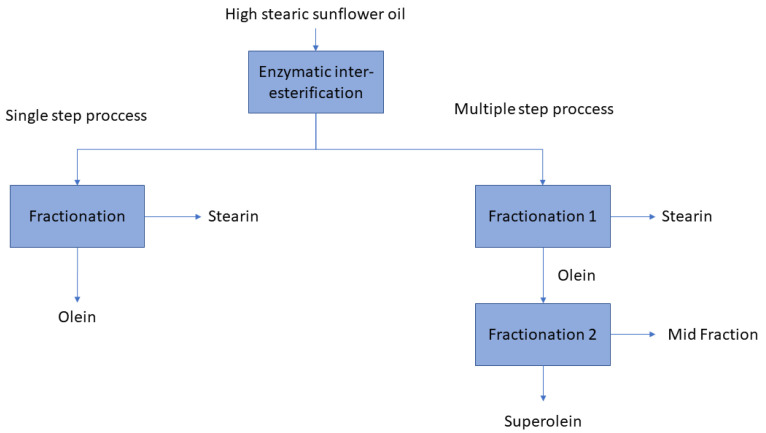
General schemes of the one-step and two-step fractionation processes carried out in the present work for oils HO20 and ML22.

Therefore, the development of these new sunflower oils represents an increase in stearic acid content, which limits the feasibility of a single-step fractionation, which yields only one type of stearin. Based on these results, a multiple-step fractionation strategy was proposed with the aim of maximizing the production of solid stearins and generating fats with a wider variety of compositions and properties, suitable for a range of formulations, as carried out with palm oil [13]. In this approach, the oils were first subjected to an initial fractionation at moderate temperatures, resulting in an olein and a stearin fraction. The resulting olein was then subjected to a second fractionation step at lower temperatures to further deplete its content of SUS-type TAGs (Figure 3). This study presents the results obtained after optimizing the process for each oil, which involved fractionations at different temperatures. In the first step, the oils were isothermally fractionated at final temperatures of 20 °C (20 °C/20 °C) for HO20 and 22 °C (22 °C/22 °C) for ML22. Under these conditions, the corresponding stearin and olein were obtained. The compositions of these fractions are shown in Table 5 and Table 6. Stearins were characterized by a high content of SSS-type TAGs: 9.4% for HO20 and 11.5% for ML22. The content of SUS + SSU-type TAGs was 27.9% in both cases, with stearic acid content between 26% and 27%. The resulting oleins still contained relatively high levels of TAGs at high melting points, with 12% and 16% SUS TAGs and stearic acid content ranging from 17% to 19%. These oleins were subsequently refractionated at lower temperatures to further reduce the content of SUS TAGs. The second fractionation step was carried out at a nucleation temperature of 15 °C and a final temperature of 12 °C for the HO20-derived olein and 10 °C for the ML22-derived olein. The products of this second fractionation were named, analogously to palm oil processing, mid-fraction and superolein (Figure 3). The compositions of these fractions are shown in Table 5 and Table 6. The mid-fractions exhibited a TAG profile different from that of the stearins, being particularly rich in SUS TAGs, with 29.9% in the case of HO20 and 36.7% for ML22, and containing low levels of SSS TAGs, making them distinct from the stearin fractions. The liquid fraction obtained in this stage was termed superolein. It was characterized by low levels of high-melting-point TAGs: 8.2% for HO20 and 6.1% for ML22. Although liquid, this fraction showed a relatively high level of saturated fatty acids, with stearic acid accounting for approximately 15%. This stearic acid was mainly distributed in monosaturated (SUU) TAGs of the SUU or USU type. The superoleins were also rich in oleic acid, comprising 73% in HO20 and 61% in ML22, indicating that these oils could offer high oxidative stability, making them suitable for high-temperature applications.

**Figure 4:**
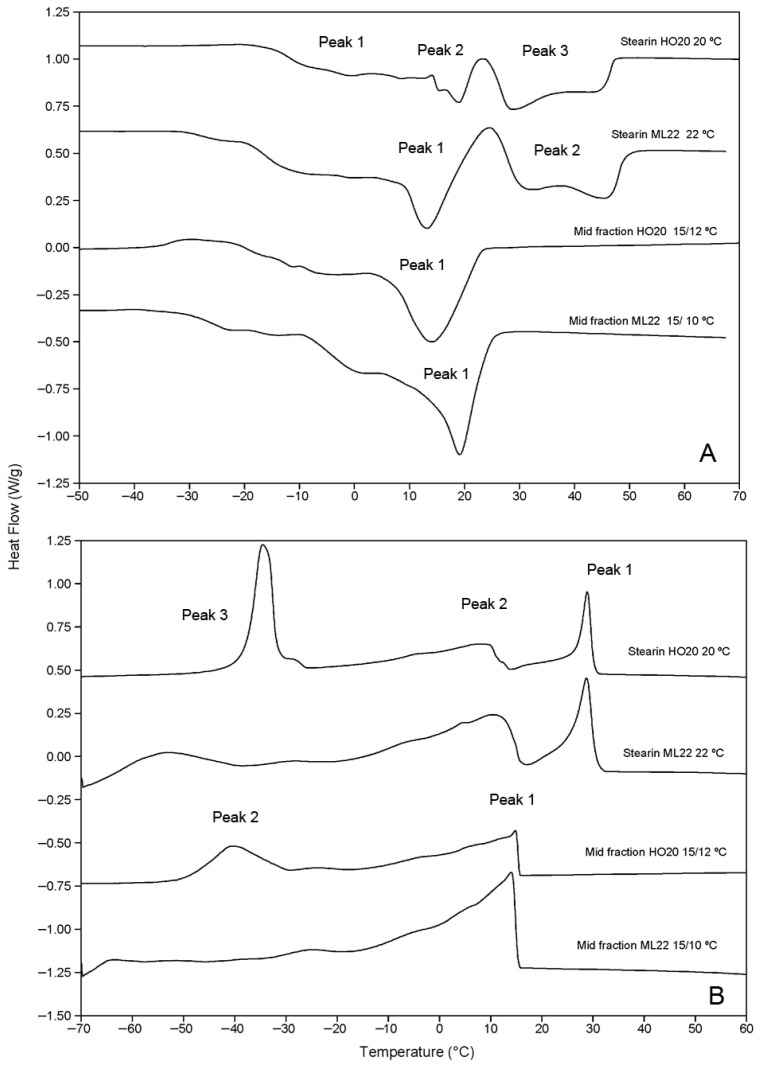
Melting (**A**) and crystallization (**B**) curves of stearins and mid-fractions produced by successive fractionation of the high-stearic sunflower oils HO20 and ML22 at the indicated temperatures. Exo up.

**Figure 5:**
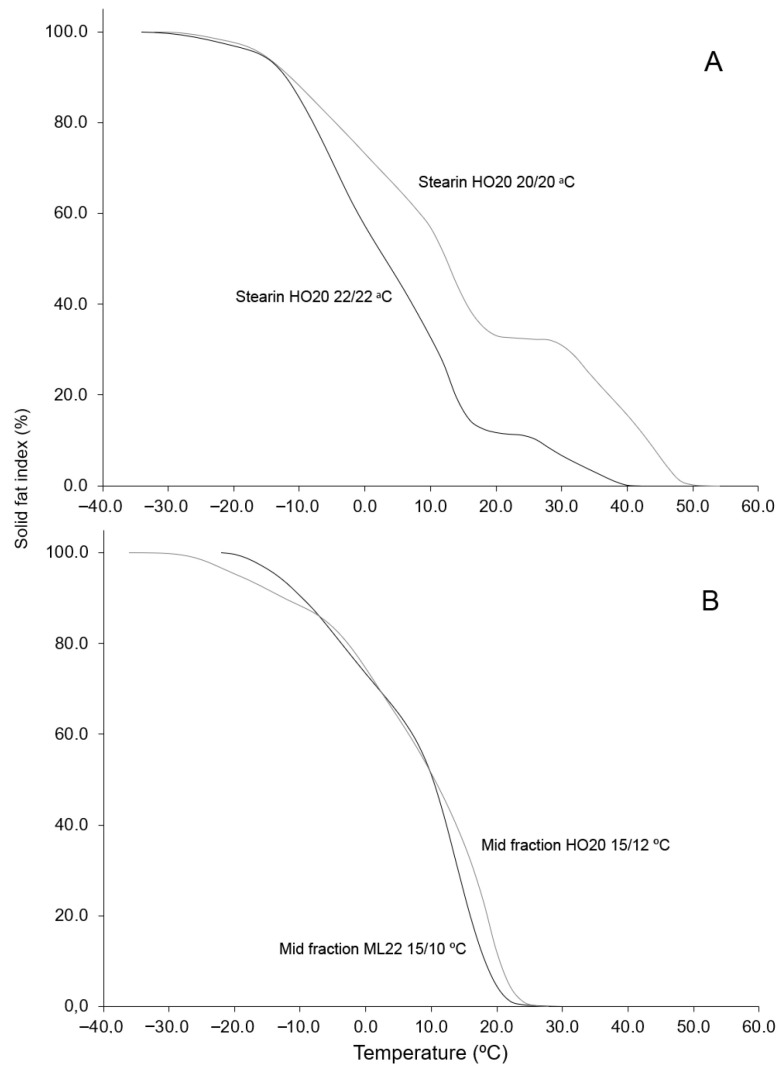
Solid fat index (SFI) curves of stearins (**A**) and mid-fractions (**B**) resulting from sequential fractionation of the oils HO20 (grey) and ML22 (black). They were obtained by continuous integration of DSC melting curves.

It is also important to highlight the process yields. The double-fractionation strategy enabled a higher yield of solid fractions in both cases. For HO20/EIE, the yields were 25.8% for stearin and 20.7% for mid-fraction, totaling 46.5% solid material compared to 25.7% obtained from single-step fractionation. In the case of ML22/EIE, the stearin yield was 23.4% and the mid-fraction yield was 29.5%, resulting in a total solid fraction of 52.9%. Thus, this oil provided higher yields of both the stearin and mid-fraction due to its higher saturation level and its ability to undergo fractionation at lower temperatures, facilitating crystallization of a larger proportion of high-melting-point TAGs. As with the stearins obtained through single-step fractionation, those generated via the multiple-step fractionation process were characterized by DSC. The melting and crystallization curves of the stearins and mid-fractions are shown in Figure 4. Stearins exhibited a very similar thermal behavior to fractions obtained through single-step fractionation, with broad melting profiles, resulting in melting ranges of 66.7 °C and 84.83 °C for the HO20 and ML22 stearins, respectively (Table 7). Both fractions showed a broad high-temperature melting peak corresponding to the SSS TAG fraction. The SFI curves obtained by integrating the melting thermograms illustrate this behavior more clearly (Figure 5). In these curves, the progressive melting profile of the stearins can be more clearly observed (Figure 5A), in contrast to the faster and more abrupt melting of the mid-fractions (Figure 5B). As noted previously for the single-step stearins, these fats may be suitable for the production of margarines, shortenings, and bakery fats, as well as substrates for the production of structured fats via interesterification. In contrast, the mid-fractions showed narrower melting profiles, with a predominant peak at 15.25 °C and 19.32 °C for HO20 and ML22, respectively (Table 7), making them more suitable for applications such as fillings and soft confectionery products [19]. In their current form, the mid-fractions produced are not suitable for the formulation of cocoa butter equivalents (CBEs), although it is possible that solvent fractionation could be applied to obtain them from those fractions [20,21].

Thus, the sequential fractionation process following EIE enables a more efficient utilization of high-stearic sunflower oils, allowing for the production of stearins rich in stearic acid with distinct melting profiles that can be tailored to a wide range of food applications. The process also significantly increases the yield of solid fractions, adding more value to high-stearic oils produced through the biotechnological modification of oilseeds. In particular, several differences between the two studied oils should be highlighted. HO20/EIE oil, with a higher content of oleic acid, produced slurries with higher viscosities compared to ML22/EIE oil, which could be fractionated at lower temperatures and produced a greater amount of solid fat. The fractions obtained from each oil exhibited similar melting profiles, although those derived from ML22 tended to show broader melting ranges, likely due to a more complex TAG composition.

## 4. Conclusions

In the present study, two high-stearic sunflower oils were used, which differed primarily in their oleic and linoleic acid contents. Both oils were enzymatically interesterified, and different approaches were explored for obtaining solid fractions through dry fractionation. Single-step fractionation was found to be limited by the high viscosity of the slurries crystallized at temperatures below 16 °C or 12 °C, depending on the oil, which hindered the separation of liquid and solid phases by filtration. The resulting stearins showed moderate levels of SSS TAGs and exhibited very broad melting ranges. These limitations were overcome by designing a multistep process consisting of an initial fractionation at moderate temperature, yielding a stearin rich in SSS TAGs and an olein, which was further fractionated at lower temperatures. This second fractionation resulted in a mid-fraction, rich in SUS TAGs and characterized by a narrower melting range, and a superolein, consisting of an oil rich in oleic acid and with a stearic acid content of approximately 15%, distributed mainly in SUU TAGs. The proposed process significantly increases the solid fat obtained from these oils, resulting in total solid fat recoveries exceeding 50% of the initial oil. The fact that the step fractionation of these oil was demonstrated to be feasible and efficient, giving high yields of stearins with different properties, could be determinant for the industrial exploitation of new sunflower lines.

## Figures and Tables

**Table 1:** Triacylglycerol species composition of the high-stearic–high-oleic oils used in this work, before and after enzymatic inter esterification (EIE).

Oil Sample	Composition (% *w*/*w*)											
	POSt	POO	StStSt	StOSt	StOO	OOO	StOL	OOL	StLL	StOA	StOB	Others
												
HO20	3.9	7.4	0.0	7.0	33.2	26.7	3.6	2.8	0.9	1.6	1.2	11.7
HO20/EIE	3.8	6.6	0.9	7.7	25.5	31.7	3.6	6.0	0.0	1.7	3.4	9.1
												
ML22	3.6	7.4	0.0	6.1	28.6	20.2	5.1	3	6.3	1.4	1.2	17.1
ML22/EIE	3.8	5	1.1	7.9	21.1	17.7	10.1	13.2	1.1	1.8	1.8	15.4

Triacylglycerol species were named using 3 letters corresponding to fatty acids: P, palmitic; St, stearic; O, oleic; L, linoleic; A, arachidic; B, behenic. Data represent the means of three technical replicates; the variation due to the method of determination was below 5%. The order given to the letters in all species do not imply any information about their distribution.

**Table 2:** Composition of the fatty acid, iodine value (IV) and triacylglycerol (TAG) classes of the high-stearic–high-oleic oils used in this work, before and after enzyme interesterification (EIE).

Oil Sample	Fatty Acids (% *w*/*w*)							TAG Classes			
	16:0	18:0	18:1	18:2	20:0	20:0	IV	SSS	SUS	SUU	UUU
HO20	5.9	19.5	67.0	4.7	1.3	1.6	65.8	0.0	16.4	53.6	30.0
HO20/EIE								1.9	17.1	43.0	38.0
ML22	6.0	22.1	54.0	13.9	1.8	2.2	70.5	0.0	15.7	57.8	26.5
ML22/EIE								2.7	18.1	45.1	34.1

In triacylglycerols, class S represents a saturated fatty acid and class U an unsaturated fatty acid. The data correspond to the means of three technical replications, and the variation due to the method of determination was below 5%. The order given to the class does not imply any information about its distribution. SSS: trisaturated TAGs; SUS: disaturated TAGs; SUU: monosaturated TAGs; UUU: triunsaturated TAGs.

**Table 3:** Composition of fatty acid and triacylglycerol (TAG) classes of the initial HO20 and ML22 oils and the stearins and oleins resulting from single-step fractionation at 16 °C and 12 °C final temperature. The yield of the stearin fraction is also included.

	Composition (% *w*/*w*)										
	Fatty Acids						Triacylglycerol Classes				Yield (%)
	16:0	18:0	18:1	18:2	20:0	20:0	SSS	SUS	SUU	UUU	
HO20/EIE	5.9	19.5	67.0	4.7	1.3	1.6	1.9	17.1	43.7	38.0	
Stearin 20 °C/16 °C	7.0 ± 0.2	32.5 ± 1.0	51.9 ± 1.2	3.0 ± 0.2	2.5 ± 0.1	3.1 ± 0.3	9.2 ^a^ ± 0.9	39.3 ^a^ ± 0.9	29.9 ^a^ ± 0.7	21.6 ^a^ ± 0.6	25.7 ^a^ ± 2.1
Olein 20 °C/16 °C	5.1 ± 0.1	15.4 ± 0.2	72.1 ± 0.2	4.6 ± 0.1	1.2 ± 0.0	1.6 ± 0.1	0.1 ± 0.1	9.8 ± 0.9	47.8 ± 0.4	42.4 ± 0.6	
											
ML22/EIE	6.0	22.1	54.0	13.9	1.8	2.2	2.7	18.1	45.1	34.1	
Stearin 22 °C/12 °C	7.3 ± 0.3	31.6 ± 1.3	44.7 ± 1.4	10.8 ± 0.4	2.4 ± 0.2	3.2 ± 0.1	8.0 ^a^ ± 0.6	38.5 ^a^ ± 4.0	32.5 ^a^ ± 1.7	21.0 ^a^ ± 2.9	38.9 ^b^ ± 2.6
Olein 22 °C/12 °C	5.2 ± 0.1	15.2 ± 0.2	61.0 ± 0.5	15.9 ± 0.2	1.0 ± 0.0	1.7 ± 0.4	0.1 ± 0.6	7.4 ± 4.0	49.2 ± 1.7	43.3 ± 2.9	

Data correspond to the average ± SD of 3 independent fractionation experiments. The composition of TAG species of fractions is shown in Supplementary Tables S1 and S2. In triacylglycerols, class S represents a saturated fatty acid and class U an unsaturated fatty acid. The data correspond to the means of three technical replications, and the variation due to the method of determination was below 5%. The order given to the class does not imply any information about its distribution. SSS: trisaturated TAGs; SUS: disaturated TAGs; SUU: monosaturated TAGs; UUU: triunsaturated TAGs. One-way analysis of variance (ANOVA) was used to compare triglyceride class composition of stearins and process yields. Superscript letters denote statistically significant differences among groups (*p* < 0.05). Groups sharing the same letter are not significantly different.

**Table 4:** Data from melting and crystallization curves obtained from stearins produced by single-step fractionation of the high-stearic sunflower oils HO20 and ML22 at 20 °C/16 °C and 22 °C/12 °C, respectively.

	Temperature (°C)					
	Onset	Offset	D	Peak 1	Peak 2	Peak 3
Melting						
Stearin HO20 20 °C/16 °C	−20.3 ± 1.0	50.6 ± 0.7	70.9 ± 1.2	8.3 ± 0.3	18.9 ± 0.6	29.6 ± 0.2
Stearin ML22 22 °C/12 °C	−34.3 ± 3.1	44.1 ± 3.9	78.4 ± 0.8	−3.4 ± 1.7	14.1 ± 2.5	29.2 ± 3.5
						
Crystallization						
Stearin HO20 20 °C/16 °C	33.2 ± 0.6			31.7 ± 0.9	17.9 ± 0.3	−30.5 ± 0.2
Stearin HO20 20 °C/16 °C	26.8 ± 4.2			23.5 ± 3.3	7.9 ± 4.7	−55.8 ± 2.3

The data correspond to the average plus or minus the standard deviation of 3 to 5 records obtained from independent fractionation operations.

**Table 5:** The composition of the fatty acid and triacylglycerol (TAG) classes of the stearins and oleins resulting from successive fractionation at 20 °C/20 °C and 15 °C/12 °C final temperature of the oil HO20. Yields based on the initial oil were also included.

	Composition (% *w*/*w*)										Yield (%)
	Fatty Acids						TAG Classes				
HO20	16:0	18:0	18:1	18:2	20:0	22:0	SSS	SUS	SUU	UUU	
Fractionation 20 °C/20 °C											
Stearin	6.9 ± 0.2	27.3 ± 1.5	57.9 ± 1.8	3.7 ± 0.1	1.9 ± 0.1	2.3 ± 0.2	9.4 ^a^ ± 1.4	27.9 ^a^ ± 1.5	35.3 ^a^ ± 2.2	27.4 ^a^ ± 0.6	25.8 ^a^ ± 1.1
Olein	5.3 ± 0.2	17.4 ± 0.9	70.1 ± 1.2	4.5 ± 0.1	1.2 ± 0.1	1.5 ± 0.0	0.1 ± 0.2	12.8 ± 1.5	46.6 ± 2.2	40.5 ± 0.6	74.2 ± 1.1
Fractionation 15 °C/12 °C											
Mid-fraction	6.3 ± 0.2	23.9 ± 0.4	62.1 ± 0.4	3.9 ± 0.0	1.7 ± 0.2	2.1 ± 0.1	0.7 ^b^ ± 0.1	29.9 ^a^ ± 3.0	41.2 ^b^ ± 0.9	28.2 ^a^ ± 2.1	20.7 ^b^ ± 1.8
Superolein	5.1 ± 0.3	14.7 ± 0.1	73.0 ± 0.5	4.8 ± 0.1	1.1 ± 0.2	1.3 ± 0.2	0.1 ± 0.1	8.2 ± 3.0	47.7 ± 0.9	44.0 ± 2.1	53.4 ± 2.9

Data correspond to the average ± SD of 3 independent fractionation experiments. The composition of the fractions of TAG species is shown in Supplementary Tables S3 and S4. In triacylglycerols, class S represents a saturated fatty acid and class U an unsaturated fatty acid. The data correspond to the means of three technical replications, and the variation due to the method of determination was below 5%. The order given to the class does not imply any information about its distribution. SSS: trisaturated TAGs; SUS: disaturated TAGs; SUU: monosaturated TAGs; UUU: triunsaturated TAGs. One-way analysis of variance (ANOVA) was used to compare triglyceride class composition of stearins and process yields. Superscript letters denote statistically significant differences among groups (*p* < 0.05). Groups sharing the same letter are not significantly different.

**Table 6:** The composition of the fatty acid and triacylglycerol (TAG) classes of the stearins and oleins resulting from successive fractionation at 22 °C/22 °C and 15 °C/12 °C final temperature of the oil ML22. Yields based on the initial oil were also included.

	Composition (% *w*/*w*)										Yield (%)
	Fatty Acids						TAG Classes				
ML22	16:0	18:0	18:1	18:2	20:0	22:0	SSS	SUS	SUU	UUU	
Fractionation 22 °C/22 °C											
Stearin	6.3 ± 0.2	26.2 ± 0.7	50.1 ± 0.6	12.0 ± 0.2	2.0 ± 0.1	3.0 ± 0.4	11.5 ^a^ ± 0.5	27.9 ^a^ ± 0.6	36.0 ^a^ ± 0.6	24.7 ^a^ ± 0.4	23.4 ^a^ ± 0.4
Olein	5.5 ± 0.1	19.3 ± 0.3	57.3 ± 0.4	14.1 ± 0.1	1.5 ± 0.1	2.3 ± 0.3	0.1 ± 0.0	16.4 ± 0.4	48.2 ± 0.4	35.2 ± 0.1	76.6 ± 0.4
Fractionation 15 °C/10 °C											
Mid-fraction	6.5 ± 0.2	27.3 ± 0.7	49.8 ± 0.6	11.5 ± 0.3	1.8 ± 0.3	3.0 ± 0.2	1.9 ^b^ ± 0.2	36.7 ^b^ ± 0.4	40.0 ^b^ ± 0.4	22.3 ^b^ ± 0.3	29.5 ^b^ ± 0.7
Superolein	4.8 ± 0.1	15.0 ± 0.7	62.1 ± 0.6	15.4 ± 0.3	1.1 ± 0.1	1.7 ± 0.2	0.0 ± 0.0	6.1 ± 0.2	52.2 ± 0.1	42.6 ± 0.1	47.1 ± 0.5

Data correspond to the average ± SD of 3 independent fractionation experiments. The species of fractional TAG composition is shown in Supplementary Table S3. In triacylglycerols, class S represents a saturated fatty acid and class U an unsaturated fatty acid. The data correspond to the means of three technical replications, and the variation due to the method of determination was below 5%. The order given to the class does not imply any information about its distribution. SSS: trisaturated TAGs; SUS: disaturated TAGs; SUU: monosaturated TAGs; UUU: triunsaturated TAGs. One-way analysis of variance (ANOVA) was used to compare triglyceride class composition of stearins and process yields. Superscript letters denote statistically significant differences among groups (*p* < 0.05). Groups sharing the same letter are not significantly different.

**Table 7:** Data from the melting and crystallization curves obtained from stearins produced by successive fractionation of the high-stearic sunflower oils HO20 and ML22 at the indicated temperatures.

	Temperature °C					
	Onset	Offset	Melting Interval	Peak 1	Peak 2	Peak 3
Melting						
Stearin HO20 20 °C/20 °C	−31.31 ± 1.15	49.67 ± 0.65	80.98 ± 1.61	−8.66 ± 0.48	1.87 ± 1.71	40.15 ± 1.23
Stearin ML22 22 °C/22 °C	−32.77 ± 0.96	52.04 ± 1.12	84.81 ± 2.06	13.38 ± 0.62	32.57 ± 0.71	
Mid-fraction HO20 15 °C/12 °C	−28.29 ± 0.07	27.88 ± 3.47	56.17 ± 3.54	−10.16 ± 0.88	15.25 ± 1.70	
Mid-fraction ML22 15 °C/10 °C	−32.52 ± 1.17	27.37 ± 1.08	59.89 ± 2.24	19.32 ± 0.63		
						
Crystallization						
Stearin HO20 20 °C/20 °C	32.25 ± 0.83			29.59 ± 0.83	10.43 ± 0.64	−35.07 ± 0.69
Stearin ML22 22 °C/22 °C	32.36 ± 1.01			28.54 ± 1.01	10.68 ± 0.85	−51.67 ± 0.85
Mid-fraction HO20 15 °C/12 °C	16.53 ± 0.87			15.02 ± 0.56	−42.27 ± 2.02	
Mid-fraction ML22 15 °C/10 °C	15.85 ± 0.40			14.22 ± 0.33		

The data correspond to the average plus or minus the standard deviation of 3 to 4 records obtained from independent fractionation operations.

## Data Availability

The original contributions presented in this study are included in the article. Further inquiries can be directed to the corresponding authors.

## Supplementary Materials

The following supporting information can be downloaded at: https://www.mdpi.com/article/10.3390/foods15101784/s1, Figure S1: General Scheme of the temperature ramps applied in the fractionations depicted in the manuscript.; Figure S2: Laboratory scale stainless steel filtration chamber used for filtration and squeezing of stearins in fractionation trials.; Table S1: Composition of triacylglycerol (TAG) of the initial HO20 oil and the stearins and oleins resulting from single step fractionation at 20 °C /16 °C.; Table S2: Composition of triacylglycerol (TAG) of the initial ML22 oil and the stearins and oleins resulting from single step fractionation at 22 °C /12 °C.; Table S3: Composition of triacylglycerol (TAG) of the initial HO20 oil and the stearins and oleins resulting from fractionation at 20 °C /20 °C.; Table S4: Composition of triacylglycerol (TAG) of the stearins and oleins resulting from fractionation at 15 °C /12 °C of the HO20 Olein 20 °C /20 °C.; Table S5: Composition of triacylglycerol (TAG) of the initial ML22 oil and the stearins and oleins resulting from fractionation at 22 °C /22 °C.; Table S6: Composition of triacylglycerol (TAG) of the stearins and oleins resulting from fractionation at 15 °C /10 °C of the ML22 Olein 22 °C /22 °C.
