# Supplementary material for: Multi-Step Fractionation of New High Stearic Sunflower Oils
Source: Foods. 2026 May 18;15(10):1784. doi: 10.3390/foods15101784 (PMC13205973; doi:10.3390/foods15101784)
Supplement: Supplementary file 1 [file foods-15-01784-s001.zip › foods-4259230-supplementary.pdf]

Supplementary material

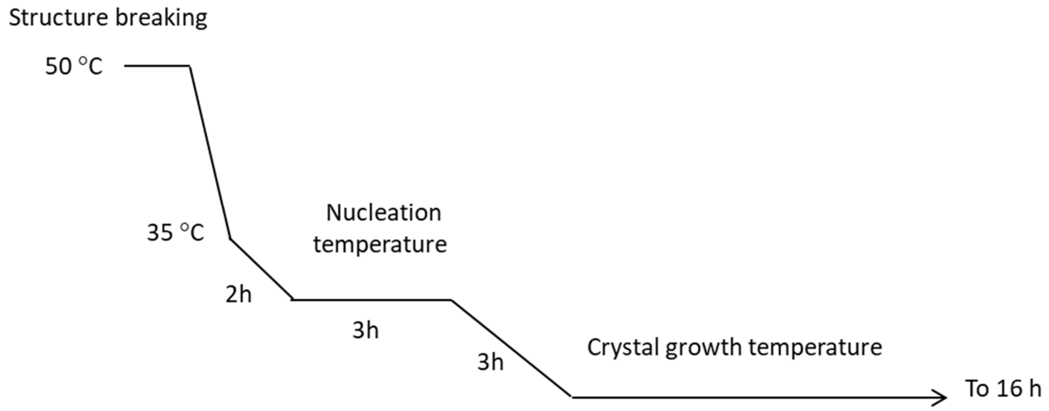

Supplementary figure S1. General Scheme of the temperature ramps applied in the fractionations depicted in the manuscript. The conditions of a fractionation will give given by the temperature of nucleation and the final crystallization temperature separated by a slash. i. e. 20 °C/ 16°C.

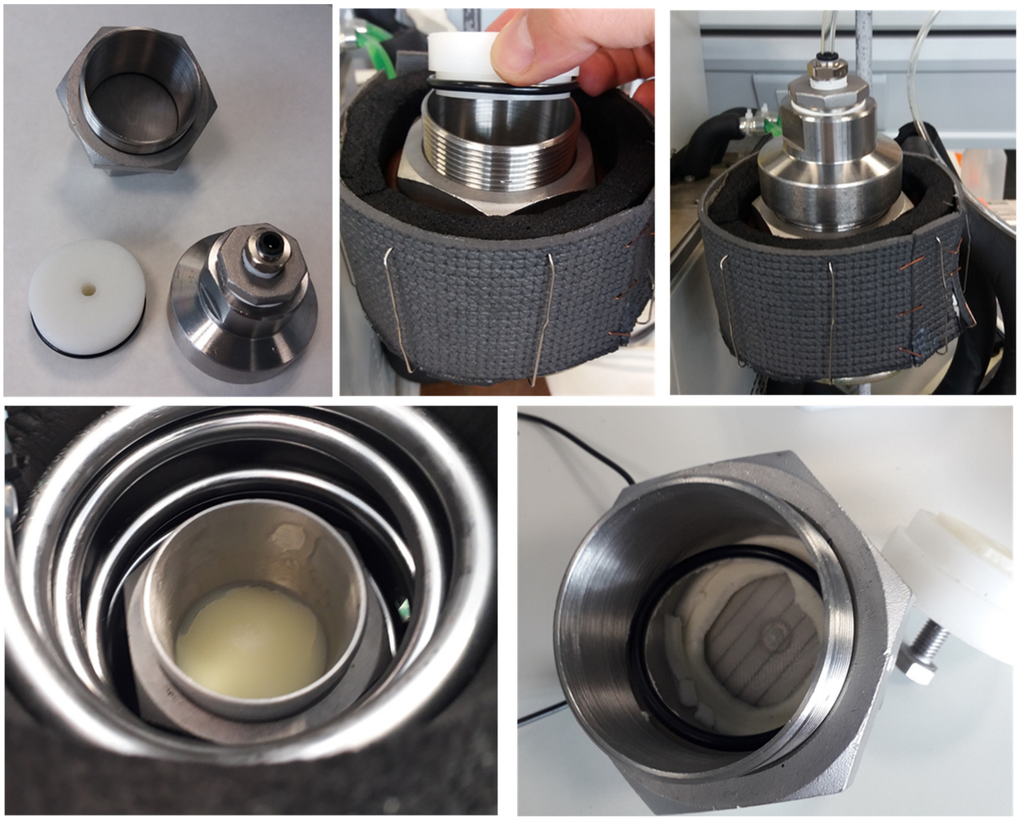

Supplementary figure S2. Laboratory scale stainless steel filtration chamber used for filtration and squeezing of stearins in fractionation trials.

Supplementary table S1. Composition of triacylglycerol (TAG) of the initial HO20 oil and the stearins and oleins resulting from single step fractionation at 20 °C /16 °C. Triacylglycerol species were named using 3 letters corresponding to fatty acids: P, palmitic; St, stearic; O, oleic; L, linoleic; A, arachidic; B, behenic. Data corresponded to the average of 3 operations, the lower row showing the standard deviation (SD). The order given to the letters in all species do not imply any information about their distribution.

**TAG**	PStP	POP	PStSt	POSt	POO	PLSt	POL	StStSt	StOSt	StOO	StLSt	OOO	StOL	OOL	OLL	StStA	StOA	OOA	OLA	StStB	StOB	OOB	OLB
HO20/EIE	0.0	0.4	0.0	2.5	8.6	0.1	0.4	0.1	3.5	32.7	0.0	41.5	1.3	2.4	0.2	0.2	0.5	0.0	0.0	0.0	0.0	2.7	0.0
HO20 Olein 20 °C /16 °C	0.0	0.6	0.0	3.0	7.8	0.4	1.0	0.0	3.7	29.2	0.0	36.0	3.7	6.4	0.0	0.0	0.9	2.4	0.0	0.0	1.1	3.6	0.0
SD	0.0	0.0	0.2	0.1	0.2	0.2	0.0	0.3	0.4	0.5	0.0	0.5	0.1	0.1	0.0	0.2	0.1	0.1	0.0	0.1	0.1	0.1	0.0
HO20 Stearin 20 °C /16 °C	0.4	0.6	1.4	5.5	5.6	0.4	0.8	2.1	13.0	24.9	0.0	25.3	2.8	4.5	0.0	0.8	3.1	1.8	0.0	0.8	3.6	2.7	0.0
SD	0.1	0.0	0.2	0.1	0.2	0.2	0.0	0.3	0.4	0.5	0.0	0.5	0.1	0.1	0.0	0.2	0.1	0.1	0.0	0.1	0.1	0.1	0.0

Supplementary table S2. Composition of triacylglycerol (TAG) of the initial ML22 oil and the stearins and oleins resulting from single step fractionation at 22 °C /12 °C. Triacylglycerol species were named using 3 letters corresponding to fatty acids: P, palmitic; St, stearic; O, oleic; L, linoleic; A, arachidic; B, behenic. Data corresponded to the average of 3 operations, the lower row showing the standard deviation (SD). The order given to the letters in all species do not imply any information about their distribution.

**TAG**	PPSt	POP	PLP	PStSt	POSt	POO	PLSt	POL	PLL	StStSt	StOSt	StOO	StLSt	OOO	StOL	OOL	StLL	OLL	StStA	StOA	LLL	OOA	StLA	OLA	LLA	StStB	StOB	OOB	StLB	OLB
Inicial	0.2	0.5	0.1	0.7	3.8	5.0	1.0	2.5	0.4	1.1	7.9	20.8	0.8	17.7	10.1	13.2	1.1	3.2	0.3	1.8	0.0	1.3	0.1	0.6	0.0	0.4	1.8	2.1	0.2	1.0
ML22 Olein 22 °C/12 °C	0.1	0.5	0.3	0.0	2.3	6.3	0.7	3.2	0.6	0.0	2.2	23.3	0.0	22.8	7.9	16.1	0.9	4.4	0.0	0.9	0.0	1.8	0.0	0.9	0.0	0.0	0.6	3.0	0.0	1.4
SD	0.0	0.0	0.0	0.2	0.6	0.3	0.1	0.2	0.0	0.2	1.8	0.7	0.4	1.3	0.5	1.3	0.4	0.3	0.1	0.4	0.0	0.1	0.1	0.1	0.0	0.1	0.5	0.1	0.1	0.1
ML22 stearin 22 °C/12 °C	0.6	0.6	0.2	2.0	6.4	3.3	1.3	1.6	0.3	2.9	17.1	15.5	3.2	10.7	7.0	8.4	0.4	1.9	1.3	3.7	0.0	1.2	0.7	0.5	0.0	1.2	4.4	1.9	0.9	0.8
SD	0.0	0.0	0.0	0.2	0.6	0.3	0.1	0.2	0.0	0.2	1.8	0.7	0.4	1.3	0.5	1.3	0.4	0.3	0.1	0.4	0.0	0.1	0.1	0.1	0.0	0.1	0.5	0.1	0.1	0.1

Supplementary table S3. Composition of triacylglycerol (TAG) of the initial HO20 oil and the stearins and oleins resulting from fractionation at 20 °C /20 °C. Triacylglycerol species were named using 3 letters corresponding to fatty acids: P, palmitic; St, stearic; O, oleic; L, linoleic; A, arachidic; B, behenic. Data corresponded to the average of 3 operations, the lower row showing the standard deviation (SD). The order given to the letters in all species do not imply any information about their distribution.

**TAG**	PStP	POP	PStSt	POSt	POO	PLSt	POL	StStSt	StOSt	SttOO	StLSt	OOO	StOL	OOL	SLL	OLL	StStA	StOA	OOA	OLA	SOB	OOB	OLB
Inicial	0.0	0.4	0.0	2.5	8.6	0.1	0.4	0.1	3.5	32.7	0.0	41.5	1.3	2.4	0.2	0.2	0.5	0.0	0.0	0.0	0.0	2.7	0.0
HO20 Olein 20 °C /20 °C	0.0	0.5	0.1	3.4	7.2	0.5	1.0	0.1	5.5	27.7	0.3	33.5	4.2	6.7	0.2	0.1	0.8	1.9	0.9	0.1	1.4	3.6	0.4
SD	0.0	0.1	0.3	0.2	0.2	0.0	0.1	0.5	1.2	0.6	0.1	0.9	0.2	0.2	0.0	0.2	0.2	0.1	0.1	0.1	0.1	0.3	0.3
HO20 Stearin 20 °C /20 °C	0.6	0.6	2.3	4.9	5.0	0.4	0.7	3.6	14.1	21.3	0.9	23.4	3.0	4.3	0.2	1.5	3.0	1.7	0.1	1.6	3.8	2.8	0.3
SD	0.0	0.1	0.3	0.2	0.2	0.0	0.1	0.5	1.2	0.6	0.1	0.9	0.2	0.2	0.0	0.2	0.2	0.1	0.1	0.1	0.1	0.3	0.3

Supplementary table S4. Composition of triacylglycerol (TAG) of the stearins and oleins resulting from fractionation at 15 °C /12 °C of the HO20 Olein 20 °C /20 °C. Triacylglycerol species were named using 3 letters corresponding to fatty acids: P, palmitic; St, stearic; O, oleic; L, linoleic; A, arachidic; B, behenic. Data corresponded to the average of 3 operations, the lower row showing the standard deviation (SD). The order given to the letters in all species do not imply any information about their distribution.

**TAG**	PStP	POP	PtSSt	POSt	POO	PLSt	POL	StStSt	StOSt	StOO	StLSt	OOO	StOL	OOL	StLL	OLL	StStA	StOA	OOA	OLA	StStB	StOB	OOB	OLB
HO20 Olein 20 °C /20 °C	0.0	0.5	0.1	3.4	7.2	0.5	1.0	0.1	5.5	27.7	0.3	33.5	4.2	6.7	0.2	0.1	0.8	1.9	0.9	0.1	1.4	3.6	0.4	0.0
HO20 Superolein 15 °C/12 °C	0.1	0.5	0.1	2.2	7.5	0.7	1.0	0.0	2.4	27.5	1.1	36.6	4.7	7.1	0.1	0.4	0.0	0.6	2.3	0.3	0.0	0.7	3.7	0.5
SD	0.0	0.0	0.0	0.6	0.3	0.2	0.1	0.0	1.5	0.5	0.1	1.9	0.2	0.3	0.1	0.1	0.1	0.4	0.0	0.1	0.0	0.5	0.3	0.0
HO20 Mid fraction 15 °C/12 °C	0.1	0.6	0.2	6.3	5.6	0.6	0.7	0.1	14.1	25.0	1.2	23.6	3.4	4.4	0.1	0.2	0.1	3.1	2.2	0.3	0.0	3.9	3.5	0.4
SD	0.0	0.0	0.0	0.6	0.3	0.2	0.1	0.0	1.5	0.5	0.1	1.9	0.2	0.3	0.1	0.1	0.1	0.4	0.0	0.1	0.0	0.5	0.3	0.0

Supplementary table S5. Composition of triacylglycerol (TAG) of the initial ML22 oil and the stearins and oleins resulting from fractionation at 22 °C /22 °C. Triacylglycerol species were named using 3 letters corresponding to fatty acids: P, palmitic; St, stearic; O, oleic; L, linoleic; A, arachidic; B, behenic. Data corresponded to the average of 3 operations, the lower row showing the standard deviation (SD). The order given to the letters in all species do not imply any information about their distribution.

TAG	PPSt	POP	PLP	PStSt	POSt	POO	PLSt	POL	PLL	StStSt	StOSt	StOO	StLSt	OOO	StOL	OOL	StLL	OLL	StStA	StOA	LLL	OOA	StLA	OLA	LLA	StStB	StOB	OOB	StLB	OLB
Inicial	0.2	0.5	0.1	0.7	3.8	5.0	1.0	2.5	0.4	1.1	7.9	20.8	0.8	17.7	10.1	13.2	1.1	3.2	0.3	1.8	0.0	1.3	0.1	0.6	0.0	0.4	1.8	2.1	0.2	1.0
ML22 Olein 22 °C /22 °C	0.0	0.8	0.3	0.1	4.4	6.7	1.0	3.1	0.5	0.1	6.4	21.9	0.7	18.7	10.2	13.4	1.1	3.2	0.0	1.4	0.0	1.3	0.2	0.5	0.0	0.0	1.1	1.8	0.2	0.8
SD	0.0	0.0	0.0	0.1	0.1	0.1	0.0	0.0	0.0	0.2	0.3	0.3	0.3	0.3	0.1	0.1	0.1	0.1	0.1	0.0	0.0	0.0	0.0	0.0	0.0	0.0	0.0	0.0	0.0	0.0
ML22 Stearin 22 °C /22 °C	1.0	0.8	0.2	3.5	5.7	4.8	1.2	2.3	0.4	4.4	13.2	16.7	1.9	13.1	7.8	9.4	0.8	2.1	1.5	2.3	0.0	1.0	0.3	0.4	0.0	1.1	2.0	1.3	0.3	0.5
SD	0.0	0.0	0.0	0.1	0.1	0.1	0.0	0.0	0.0	0.2	0.3	0.3	0.3	0.3	0.1	0.1	0.1	0.1	0.1	0.0	0.0	0.0	0.0	0.0	0.0	0.0	0.0	0.0	0.0	0.0

Supplementary table S6. Composition of triacylglycerol (TAG) of the stearins and oleins resulting from fractionation at 15 °C /10 °C of the ML22 Olein 22 °C /22 °C. Triacylglycerol species were named using 3 letters corresponding to fatty acids: P, palmitic; St, stearic; O, oleic; L, linoleic; A, arachidic; B, behenic. Data corresponded to the average of 3 operations, the lower row showing the standard deviation (SD). The order given to the letters in all species do not imply any information about their distribution.

TAG	PPSt	POP	PLP	PStSt	POSt	POO	PLSt	POL	PLL	StStSt	StOSt	StOO	StLSt	OOO	StOL	OOL	StLL	OLL	StStA	StOA	LLL	OOA	StLA	OLA	LLA	StStB	StOB	OOB	StLB	OLB
ML22 Olein 22 °C /22 °C	0.0	0.8	0.3	0.1	4.4	6.7	1.0	3.1	0.5	0.1	6.4	21.9	0.7	18.7	10.2	13.4	1.1	3.2	0.0	1.4	0.0	1.3	0.2	0.5	0.0	0.0	1.1	1.8	0.2	0.8
ML22 Superolein 15 °C/10 °C	0.0	0.6	0.3	0.0	1.7	7.6	0.6	3.8	0.6	0.0	1.1	22.7	0.2	22.3	11.7	16.3	1.3	3.9	0.0	0.0	1.4	1.3	0.0	0.6	0.0	0.0	0.2	1.8	0.0	0.9
SD	0.0	0.0	0.0	0.1	0.1	0.1	0.0	0.0	0.1	0.1	0.3	0.2	0.1	0.1	0.1	0.2	0.1	0.0	0.0	0.0	0.0	0.0	0.0	0.0	0.0	0.0	0.1	0.0	0.0	0.0
ML22 Mid fraction 15 °C/10 °C	0.1	1.1	0.3	0.2	8.7	4.8	2.0	2.1	0.3	0.1	15.0	19.8	3.5	11.8	8.4	8.5	0.7	2.0	0.0	1.5	2.2	1.3	0.6	0.4	0.0	0.0	2.7	1.7	0.6	0.6
SD	0.0	0.0	0.0	0.1	0.1	0.1	0.0	0.0	0.1	0.1	0.3	0.2	0.1	0.1	0.1	0.2	0.1	0.0	0.0	0.0	0.0	0.0	0.0	0.0	0.0	0.0	0.1	0.0	0.0	0.0
